# Amyopathic Dermatomyositis Presenting With Oral Mucocutaneous Lesion and No Muscle Involvement in an Elderly Woman: A Mimicry of Fungal Infection and Eczema

**DOI:** 10.7759/cureus.92739

**Published:** 2025-09-19

**Authors:** Satya Rijal, Aishwarya Joshi, Richa Tikaria, P. Khanal, Nasatya Khadka

**Affiliations:** 1 Internal Medicine, University of Michigan Health-Sparrow Hospital, East Lansing, USA; 2 Internal Medicine, Michigan State University College of Human Medicine, East Lansing, USA; 3 Internal Medicine, Emilio Aguinaldo College, Manila, PHL

**Keywords:** amyopathic dermatomyositis, ivig therapy, no muscle weakness, oral mucosal lesions, skin manifestation

## Abstract

Amyopathic dermatomyositis (ADM) is a rare subtype of dermatomyositis defined by characteristic cutaneous findings without clinical or laboratory evidence of muscle involvement for at least six months. Although lacking myopathy, patients remain at risk for systemic complications, particularly rapidly progressive interstitial lung disease (RP-ILD) in the presence of anti-melanoma differentiation-associated gene 5 (MDA5) antibodies, as well as autoimmune overlap and malignancy. Diagnostic recognition may be delayed due to subtle clinical features and non-specific biopsy results. We report a 76-year-old woman who presented with violaceous papules on the upper and lower extremities, a painless blister on the lateral tongue, and purplish discoloration of the right great toe. Initial biopsy of the oral lesion was non-specific, and differential diagnoses included eczema and fungal infection. Her symptoms persisted despite empiric topical steroids and antifungal therapy. Repeat biopsies and exclusion of muscle disease confirmed ADM. Systemic corticosteroids and hydroxychloroquine were initiated, followed by mycophenolate mofetil, with limited response. The addition of intravenous immunoglobulin (IVIG) resulted in gradual improvement, leading to complete resolution of cutaneous lesions after 18 months, without systemic complications. This case illustrates the atypical presentation of ADM and the challenges in establishing an early diagnosis when muscle involvement is absent. It highlights IVIG as an effective option for treatment-resistant disease and underscores the importance of maintaining a high index of suspicion, pursuing thorough diagnostic evaluation, and ensuring long-term monitoring for interstitial lung disease and malignancy, even in patients who present with isolated cutaneous findings.

## Introduction

Dermatomyositis (DM) is a heterogeneous autoimmune disease classified into several subtypes, including classic DM, juvenile or adult onset, and clinically amyopathic DM (CADM) [1]. CADM, which accounts for approximately 20% of all DM cases, is characterized by the hallmark skin manifestations of dermatomyositis without clinical evidence of muscle weakness [2,3].​ While various autoantibodies are associated with DM, anti-melanoma differentiation-associated gene 5 (MDA5) autoantibodies are notably linked to skin rashes and interstitial lung disease (ILD), particularly in CADM [3]. Other myositis-specific antibodies (MSAs) and myositis-associated antibodies (MAAs), such as anti-Jo-1, anti-Mi-2, anti-TIF-1γ, and anti-NXP2, help characterize different DM phenotypes, including those with an increased risk of malignancy [3].

Despite the absence of muscle involvement, ADM shares complex immunopathological mechanisms with classic dermatomyositis, involving immune-mediated damage, environmental triggers, and genetic predispositions [3,4]. Epidemiological data suggest a higher prevalence of specific ADM subtypes, like anti-MDA5 positive CADM, in specific geographic regions, notably Southeast Asia, which highlights the potential influence of environmental factors in developing this condition [4].

Furthermore, dermatomyositis, including its amyopathic form, has a documented association with an increased risk of malignancy and interstitial lung disease, both of which can significantly impact patient prognosis and survival [4]. Given these associations, early recognition is therefore critical for timely diagnosis, appropriate management, and screening for complications [4].

## Case presentation

A 76-year-old woman with a history of celiac disease presented to dermatology with a six-month history of diffuse, violaceous, non-pruritic, and photosensitive papules. The lesions were distributed along the ulnar and radial aspects of the left forearm, the dorsal aspects of both hands, and the lower limbs. Concurrently, she reported dark purple discoloration of the right great toe and a single, painless, 5 mm blister on the left lateral tongue. The blister, initially tense with clear fluid, ruptured spontaneously after several weeks, leaving a raw erythematous base with loss of epithelial surface.

The skin lesions, including the oral blister, were unresponsive to topical clobetasol and miconazole ointment, which had been prescribed sequentially for presumed eczema and fungal infection. She denied muscle weakness, myalgia, fatigue, dysphagia, systemic symptoms, or prior history of malignancy, autoimmune diseases, or drug exposure.

**Figure 1:**
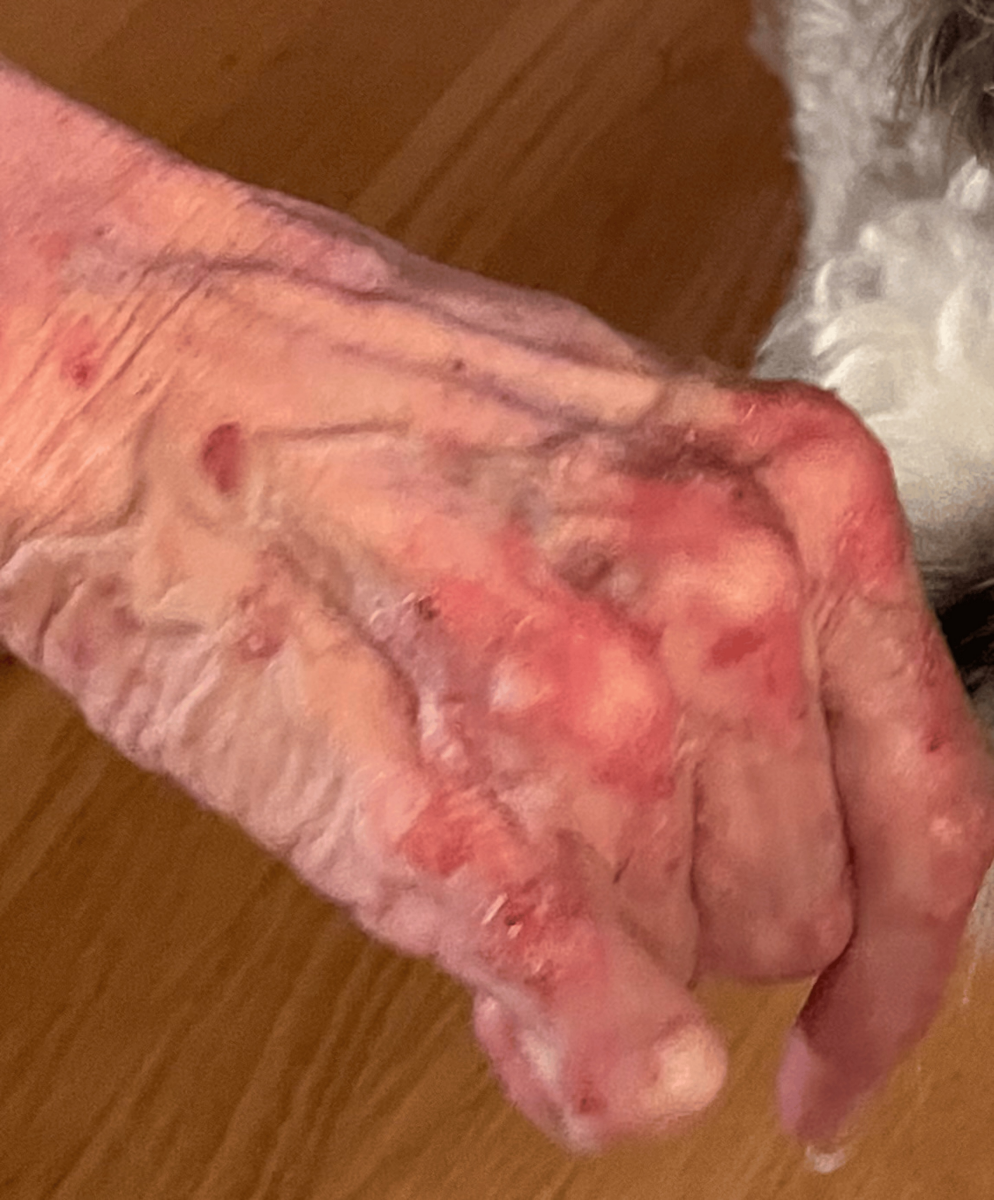
Clinical image showing characteristic violaceous Gottron's papules on the dorsum of the right hand.

**Figure 2:**
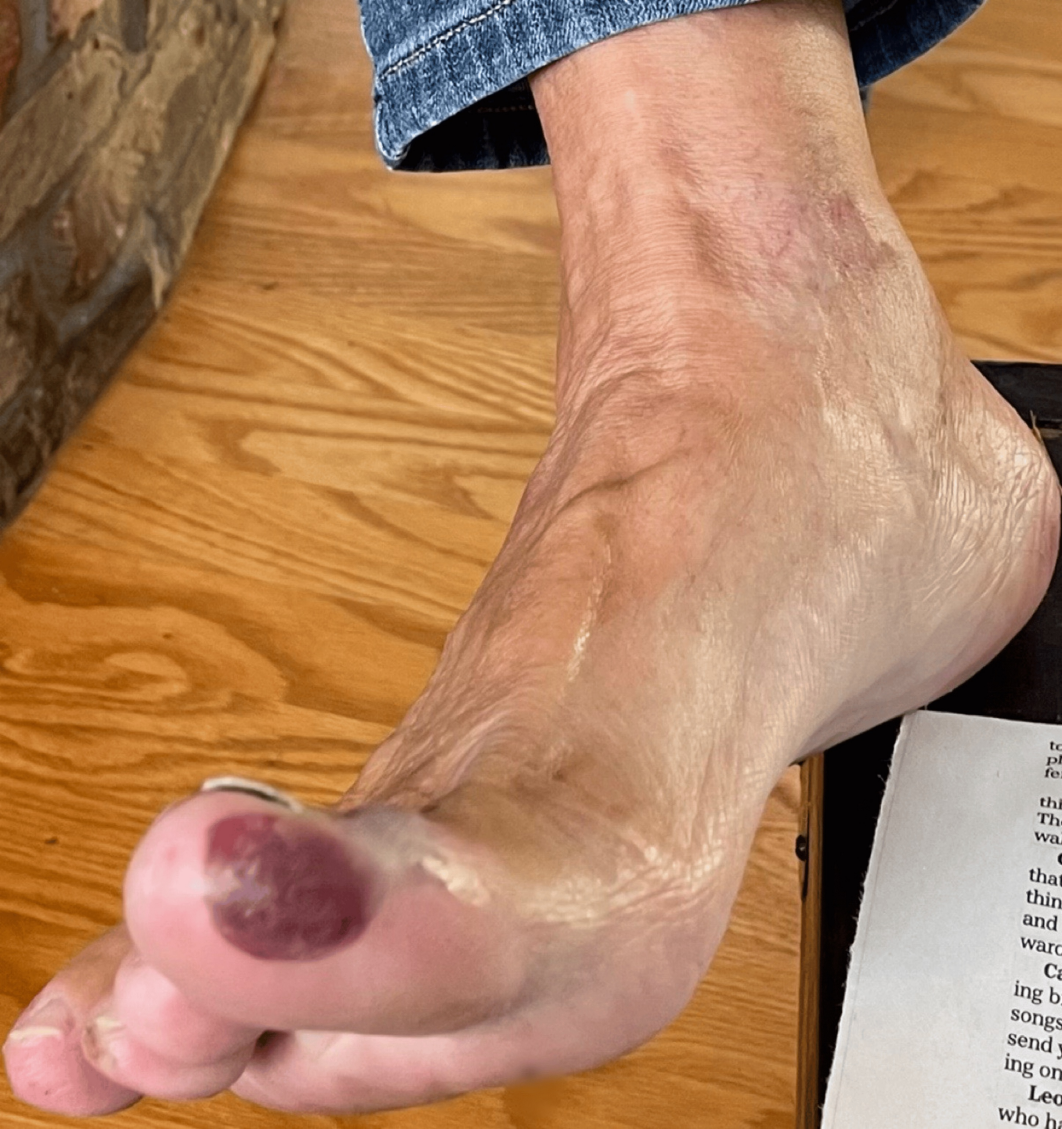
Clinical image showing purple discoloration on the plantar lateral aspect of the right great toe.

**Figure 3:**
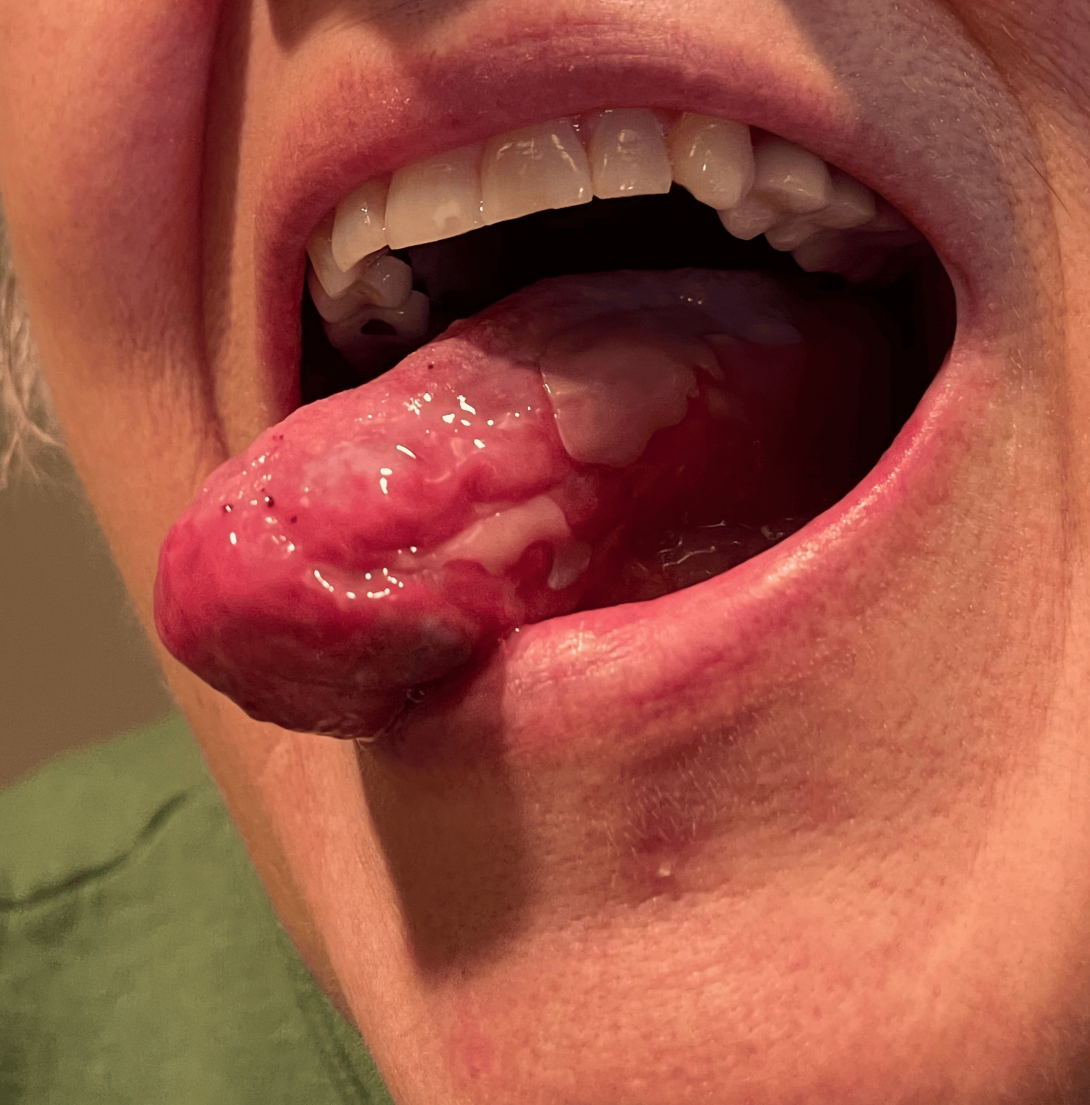
Burst blister of 5 mm in diameter located on the middle third of the left lateral tongue.

Examination revealed Gottron's papules over the dorsal hands and knuckles, a photosensitive rash involving the upper chest, back, and arms, a ruptured tongue blister, and violaceous discoloration of the right great toe (Figures 1-3). The perilesional tissue surrounding the ruptured blister appeared mildly erythematous and edematous, but no additional oral lesions were identified. Manual muscle testing was normal, with no tenderness, atrophy, or joint swelling.

**Figure 4:**
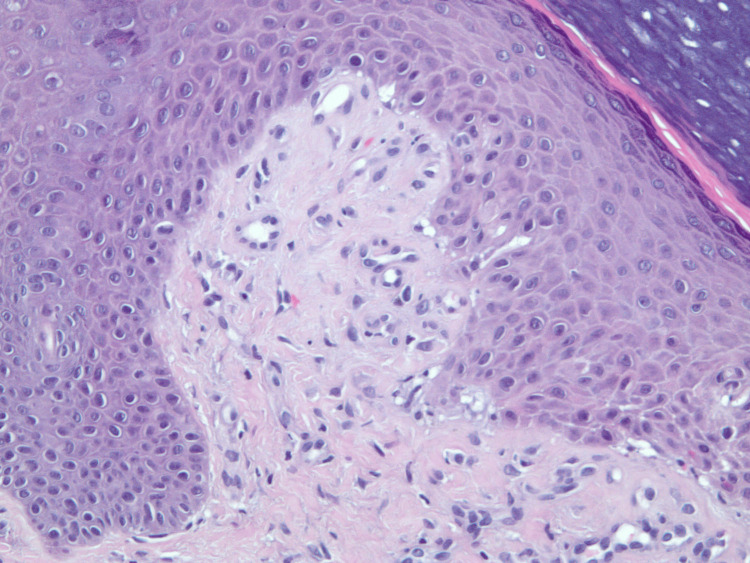
A skin biopsy from the left proximal index finger: 20x magnification view of amyopathic dermatomyositis with H&E stain revealing chronic lichenoid/interface dermatitis.

**Figure 5:**
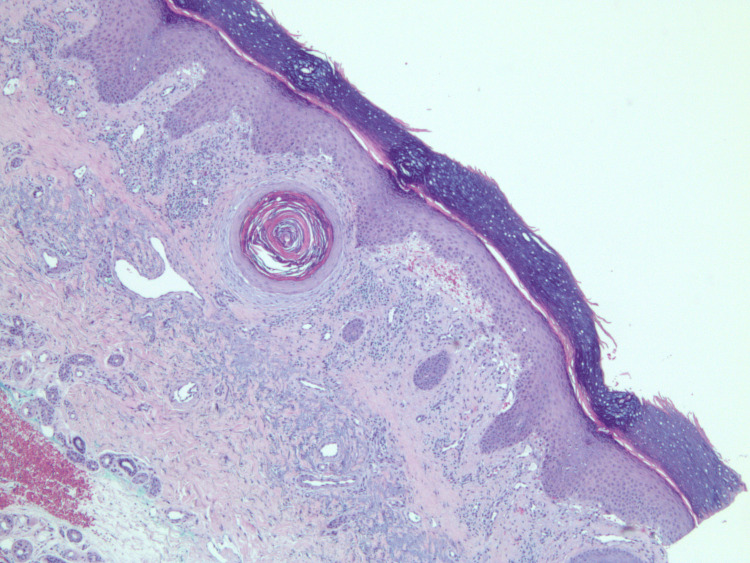
A skin biopsy from the left proximal index finger: 10x magnification view of amyopathic dermatomyositis with H&E stain displaying lichenoid mononuclear cells infiltrate that includes mostly mature lymphocytes with surrounding telangiectatic blood vessels and retractive subepidermal cleft formation that includes extravasation of red blood cells.

An initial biopsy of the tongue blister demonstrated non-specific inflammation, without diagnostic features. Following persistent disease, a repeat biopsy from the left proximal index finger was obtained. On re-examination of the archival 2016 slide, the biopsy demonstrated chronic interface dermatitis consistent with dermatomyositis. The infiltrate showed no evidence of periadnexal distribution, a feature sometimes seen in lupus erythematosus; however, there was no evidence of atypia or vasculitis. Although the age of the slide limited image clarity, the histopathologic features remain interpretable and supportive of the clinical diagnosis (Figures 4, 5).

The laboratory findings showed normal levels of aldolase, anti-melanoma differentiation-associated gene 5 (anti-MDA5) antibody, and creatine kinase (CK). The normal anti-MDA5 level suggested an atypical presentation of ADM, as this antibody is commonly associated with the condition. The patient's creatine phosphokinase (CPK) level was 165 U/L, within the normal reference range of 24-170 U/L. Lactate dehydrogenase (LDH) was also within the normal range at 249 U/L (reference range 94-250 U/L), which helped rule out significant muscle involvement. Aldolase was noted to be 6 U/L, below the reference range of <8.1 U/L. The patient exhibited signs of systemic inflammation with an elevated erythrocyte sedimentation rate (ESR) of 40 mm/h, surpassing the normal range of 0-20 mm/h, and the C-reactive protein (CRP) level of 8 mg/dL, higher than the reference range of 0-1 mg/dL.

Regarding autoantibodies, several key markers were assessed to differentiate ADM from other autoimmune conditions. The anti-MDA5 antibody level was 11 U, which is below the reference range of <20 U. While often associated with certain DM phenotypes, its low level here suggests an atypical ADM presentation. Anti-TIF-1γ antibody was 18 AU, falling below the reference range of <32 AU. The anti-NXP2 marker, typically associated with myositis, was negative. Both anti-Jo-1 antibodies (9 AU/mL, reference range <11 AU/mL) and EJ antibodies (6 AU/mL, reference range <11 AU/mL) were below their respective reference ranges, effectively ruling out anti-synthetase syndrome (Table 1). 

**Table 1:** The relevant laboratory findings at the presentation. Anti-MDA5: anti-melanoma differentiation-associated gene 5.

Test	Patient value	Reference range	Unit
Erythrocyte sedimentation rate (ESR)	40	0–20	mm/hr
C-reactive protein (CRP)	8	0–1	mg/dL
Antinuclear antibodies (ANA)	Negative	Negative	–
Creatine phosphokinase (CPK)	165	24–170	U/L
Vitamin D	20	>30	ng/mL
Lactate dehydrogenase (LDH)	249	94–250	U/L
Aldolase	6	<8.1	U/L
Jo-1 antibodies	9	<11	AU/mL
EJ antibodies	6	<11	AU/mL
Anti-MDA5	11	<20	U
Anti-TIF1-γ	18	<32	AU
Anti-NXP2	Negative	Negative	U/mL

Electromyography and MRI of the thighs revealed no evidence of myopathy, supporting a diagnosis of clinically amyopathic dermatomyositis (ADM). Imaging and malignancy work-up included chest X-ray, abdominal ultrasound, and CT scan, all of which were unremarkable. High-resolution CT of the chest showed no interstitial lung disease. Baseline cancer screening, including mammography and colonoscopy, was negative.

The patient was started on oral prednisone (1 mg/kg/day) and hydroxychloroquine (200 mg BID). After six months, her disease worsened with new violaceous papules on the torso and face, increased pruritus (7/10), and a 15% rise in Cutaneous Lupus Erythematosus Disease Area and Severity Index (CLASI) activity score [5]. Mycophenolate mofetil (1 g BID) was added, but over the next six months, she continued to deteriorate with further rash progression, fingertip and toe involvement, worsening pain (8/10), and a 20% CLASI increase.

Given refractory disease, intravenous immunoglobulin (IVIG) therapy was initiated in addition to ongoing immunosuppression. Initially, IVIG was administered over five consecutive days, divided into two equal doses infused continuously for up to 5.5 hours. The regimen was then transitioned to 2 g/kg every four weeks, later adjusted to 800 mg/kg every three to four weeks.

Over 12 months of IVIG, her skin lesions steadily improved, with complete resolution achieved after 18 months. She remained under follow-up every three months, with ongoing malignancy surveillance (mammography and colonoscopy), all negative to date. She was advised strict sun protection due to persistent photosensitivity.

## Discussion

This case highlights the successful management of an atypical presentation of amyopathic dermatomyositis (ADM) in an elderly patient. While cutaneous hallmarks such as Gottron's papules and heliotrope rash typically support diagnosis, oral involvement is distinctly uncommon. Our patient presented with a lateral tongue blister and violaceous discoloration of the great toe, both of which are atypical findings in ADM. Oral mucosal involvement is rarely reported and has been described as vesiculobullous eruptions, ulcerations, erosions, gingival erythema, telangiectasias, mucosal atrophy, edema, and leukoplakia-like areas [6-8]. A blister localized to the tongue, in the absence of more widespread oral disease, is particularly unusual and complicates the initial diagnostic impression, as such features may mimic autoimmune blistering disorders [6-8].

The cutaneous manifestations of dermatomyositis overlap with several autoimmune and inflammatory conditions, including systemic lupus erythematosus (SLE), cutaneous lupus erythematosus (CLE), mixed connective tissue disease (MCTD), Sjögren syndrome, systemic sclerosis, psoriasis, lichen planus, drug-induced eruptions, and photosensitive dermatoses [9]. In our patient, SLE and CLE were considered due to the presence of photosensitive rashes and dermal mucin deposition, but the absence of systemic involvement and negative ANA testing argued against lupus [9,10]. Sjögren syndrome was excluded based on the absence of sicca symptoms and negative serologies (anti-SSA/SSB, ANA, and rheumatoid factor). Similarly, systemic sclerosis was ruled out in the absence of Raynaud's phenomenon and skin thickening [10,11].

The diagnosis of ADM was ultimately established based on the Sontheimer criteria and the EuroMyositis registry [12], which rely on characteristic skin features, histopathological findings, and absence of muscle disease on clinical, laboratory, and imaging evaluation. Although anti-MDA5 and anti-Jo-1 antibodies are frequently implicated in distinct dermatomyositis phenotypes, our patient was seronegative, consistent with reported cases of antibody-negative ADM [13]. This underscores the importance of integrating clinical findings, repeat biopsy results, and exclusion of mimicking conditions in reaching a definitive diagnosis, especially in seronegative presentations.

The management of ADM generally begins with systemic corticosteroids and steroid-sparing immunosuppressants such as hydroxychloroquine and mycophenolate mofetil [13,14]. However, up to 20-30% of dermatomyositis patients fail to respond adequately to corticosteroids, and a subset remain refractory despite combination therapy [13,14]. Our patient demonstrated progressive cutaneous disease despite prolonged therapy with prednisone, hydroxychloroquine, and mycophenolate mofetil, highlighting the therapeutic challenge of refractory ADM.

In such cases, intravenous immunoglobulin (IVIG) has emerged as an effective treatment. IVIG exerts broad immunomodulatory effects and is increasingly used as salvage therapy in dermatomyositis [15,16]. A phase 3 randomized controlled trial demonstrated a 78.7% response rate with IVIG compared to 43.8% with placebo, with significant improvements in both muscle strength and skin manifestations [15,16]. In our patient, IVIG therapy resulted in gradual improvement and complete resolution of cutaneous disease over 18 months, without adverse effects.

This case emphasizes three important points: first, that ADM can present with atypical mucocutaneous features such as isolated tongue blisters; second, that diagnosis requires careful exclusion of mimicking autoimmune and inflammatory conditions, particularly when antibody profiles are negative; and third, that IVIG is a safe and effective therapeutic option in refractory ADM, even in elderly patients. The durable remission observed in our case supports the role of IVIG as a viable long-term potentially safe and effective long-term therapy for complex and resistant presentations of seronegative ADM.

## Conclusions

This case highlights a rare presentation of amyopathic dermatomyositis (ADM) with an isolated tongue blister and acral discoloration, features that are seldom reported and can obscure diagnosis. The absence of myositis-specific autoantibodies further complicated early recognition, underscoring the need to consider ADM even in seronegative patients with atypical mucocutaneous findings. Standard immunosuppressive therapies failed to control disease activity, but escalation to intravenous immunoglobulin (IVIG) achieved complete and sustained remission. This outcome emphasizes that IVIG can serve as an effective salvage therapy in elderly patients with refractory ADM and unusual oral involvement, leading to long-term disease control without systemic complications.
